# Berberrubine Phosphate: A Selective Fluorescent Probe for Quadruplex DNA

**DOI:** 10.3390/molecules26092566

**Published:** 2021-04-28

**Authors:** Peter Jonas Wickhorst, Heiko Ihmels

**Affiliations:** Department of Chemistry-Biology, University of Siegen, Center of Micro- and Nanochemistry and Engineering (Cµ), Adolf-Reichwein-Str. 2, 57068 Siegen, Germany; peter.wickhorst@student.uni-siegen.de

**Keywords:** alkaloids, zwitterion, light-up probe, DNA ligand, G4-DNA, quadruplex DNA

## Abstract

A phosphate-substituted, zwitterionic berberine derivative was synthesized and its binding properties with duplex DNA and G4-DNA were studied using photometric, fluorimetric and polarimetric titrations and thermal DNA denaturation experiments. The ligand binds with high affinity toward both DNA forms (*K*_b_ = 2–7 × 10^5^ M^−1^) and induces a slight stabilization of G4-DNA toward thermally induced unfolding, mostly pronounced for the telomeric quadruplex **22AG**. The ligand likely binds by aggregation and intercalation with ct DNA and by terminal stacking with G4-DNA. Thus, this compound represents one of the rare examples of phosphate-substituted DNA binders. In an aqueous solution, the title compound has a very weak fluorescence intensity (*Φ*_fl_ < 0.01) that increases significantly upon binding to G4-DNA (*Φ*_fl_ = 0.01). In contrast, the association with duplex DNA was not accompanied by such a strong fluorescence light-up effect (*Φ*_fl_ < 0.01). These different fluorimetric responses upon binding to particular DNA forms are proposed to be caused by the different binding modes and may be used for the selective fluorimetric detection of G4-DNA.

## 1. Introduction

Among the various methods used for the detection of analytes in biological systems, small probe molecules that change their photophysical properties upon interaction with the analyte are particularly useful as they may be employed to detect biomolecules even in living organisms and may thus provide real-time information about biological processes [1,2]. Along these lines, fluorescent probes that either change their emission energy (color) or fluorescence quantum yield (intensity) upon association with a target molecule are of special interest [3,4,5]. In this context, nucleic acids are an important target class of analytes because they are relevant in many biological processes [6,7] and are therefore a key target in the treatment of several diseases [8,9,10]. Besides the detection of double-stranded DNA, there is also growing interest for the detection of noncanonical DNA structures like G-quadruplex DNA (G4-DNA) since they are relevant for biological activities such as gene transcription [11,12,13,14] or telomerase inhibition [15]. Consequently, many fluorescent probes have been reported that target DNA with high selectivity and efficiency in vitro and in vivo [16,17,18,19,20,21,22,23,24,25,26,27]. Among the many different classes used for the development of DNA-sensitive fluorescent probes, natural products like sanguinarine, coralyne, or berberine (**1a**) are particularly well suited as starting points because of their combination of favorable photophysical properties with a high biocompatibility and high affinity toward G4-DNA [28,29]. In fact, many fluorescent light-up probes based on these natural products have been developed that show a highly selective increase in their emission intensity upon binding to G4-DNA [20,21,22,23,24,25,26,27,28,29,30,31,32,33,34]. Among the natural products, berberine and its derivatives are extensively studied substrates because the biocompatibility of this class of compounds has been demonstrated with their application in the treatment of various diseases [35,36,37,38]. Moreover, these compounds exhibit a light-up effect upon binding to polyanionic analytes like DNA, which is, however, rather unselective for the parent berberine [28,33,39,40]. Since the fluorescence of berberine is dependent on the polarity of the environment [41,42], the light-up effect upon complexation to DNA is significantly influenced by the binding mode of the ligand. As a result, the introduction of substituents that lead to particular binding modes of berberine derivatives to different DNA forms should enable a more selective light-up effect of the bound ligand. In this context, we focused our attention to anionic substituents. Based on the observation that anions can coordinate to specific binding sites of DNA [43,44,45,46], we reasoned that a substitution of berberine with anionic substituents may also result in particular binding modes, which in turn would cause distinct light-up effects. However, to the best of our knowledge, the DNA-binding properties of only a few DNA ligands with anionic substituents have been investigated and it has been found that these ligands exhibit a negligible affinity toward double-stranded DNA, which is supposedly caused by the strong repulsion between the negatively charged ligands and the phosphate backbone of the DNA [47,48,49]. Thus, these ligands obviously do not exhibit a significant affinity to the anion binding sides of the DNA mentioned above. Nevertheless, the positively charged nitrogen atom of the isoquinolinium unit of berberine may compensate at least the negative charge and provide an overall charge-neutral substrate. In fact, a zwitterionic anthraquinone has already been reported that exhibits a similar binding affinity to DNA as compared to its cationic parent system [50]. Along the same lines, porphyrine and phthalocyanine ligands with negatively charged substituents, with hemin as the most prominent example, show a high affinity and selectivity toward G4-DNA [51,52,53,54,55,56]. Therefore, it may be concluded that under particular circumstances the G4-DNA tolerates a negative charge of the ligand. In this context, we propose that a berberine derivative with an anionic substituent may also exhibit some special DNA-binding properties based on the combination of the different structural properties, which in turn should lead to particular optical responses of the DNA-bound ligand. Hence, we synthesized a phosphate-substituted zwitterionic berberine derivative, namely 9-*O*-Phosphonatoberberrubin (**2**), and investigated its interactions with duplex and quadruplex DNA with a special focus on fluorimeric DNA detection.

## 2. Results and Discussion

### 2.1. Synthesis

**Scheme 1:**
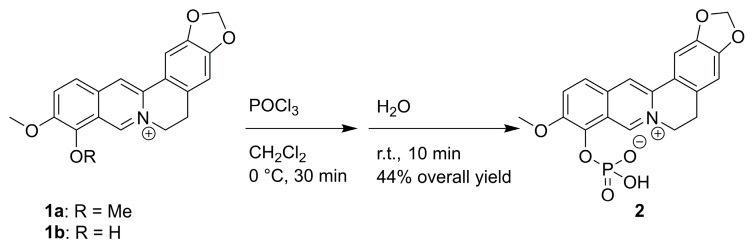
Synthesis of 9-*O*-Hydrogenphosphonatoberberrubin (**2**).

The 9-*O*-Phosphonatoberberrubin (**2**) was synthesized from berberrubine (**1b**) in a two-step synthesis (Scheme 1). In the first step, the reaction of berberrubine (**1b**) with POCl_3_ gave an intermediate phosphoryl dichloride that was directly hydrolyzed to obtain the product **2** with a 44% overall yield. The NMR-spectroscopic analysis (^1^H, ^13^C, COSY, HSQC, HMBC), elemental analysis, and mass-spectrometric data were in accordance with the structure assignment of product **2**.

### 2.2. Absorption and Emission Properties

The absorption and emission properties of compound **2** could only be tested in a few selected solvents because of its low solubility, especially in nonpolar solvents. This derivative exhibited similar absorption and emission properties as the parent berberine (**1a**) [41] with only a negligible effect from the solvent. Thus, the absorption maxima of the long-wavelength bands range between 424 nm (aqueous buffer) and 431 nm (MeOH, EtOH, 1-PrOH, MeCN, Table 1, Figure S1). However, the near-UV absorption bands between 346 nm (aqueous buffer) and 350 nm (MeOH) are slightly red-shifted as compared to **1a** (MeOH: ∆*λ* = 10 nm). At the same time, the derivative **2** has only a very weak emission with maxima between 531 nm (1-PrOH) and 558 nm (MeOH). The fluorescence quantum yields are generally higher in less polar solvents (e.g., 1-PrOH: *Φ*_fl_ = 0.04) as compared to polar protic solvents (aqueous buffer: *Φ*_fl_ < 0.01), which was also found for the parent berberine (**1a**) and may be interpreted as a result of a charge transfer (CT) in the excited state [41,42]. Usually, a CT is closely accompanied by conformational changes that may cause a symmetry-forbidden transition between the CT-excited state and the ground state [57]. Specifically, the oscillator strength of the CT state is very low, which in turn decreases the probability for emission from the CT state. Since the CT state is more stabilized in polar solvents, the fluorescence quantum yields are especially low in these solvents [57,58].

Overall, the resemblance of the absorption and emission properties of the berberine derivative **2** with the ones of the parent compound **1a** show that the phosphate group has essentially no special impact on the electronic transitions of the berberine unit as compared with the regular methoxy substituent of **1a**.

**Figure 1:**
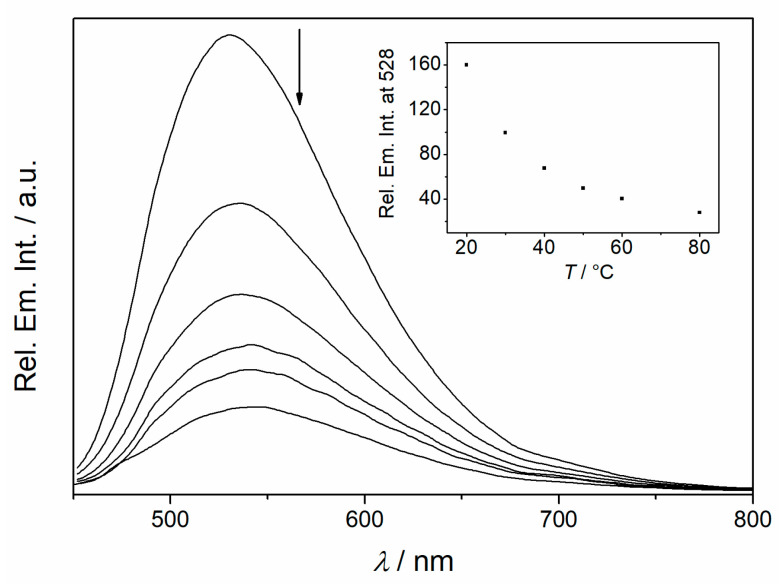
Emission spectra of **2** (*c* = 10 µM, with 0.25% *v*/*v* DMSO) in glycerol at 20 °C, 30 °C, 40 °C, 50 °C, 60 °C, and 80 °C; *λ*_ex_ = 430 nm. The arrows indicate the changes in the emission intensity with increasing temperature. Inset: Plot of the relative emission intensity at 528 nm versus the temperature of the solution.

To gain further insight into the factors that cause the weak fluorescence of compound **2**, the dependence of the emission on the viscosity of the solvent was studied using measurements in glycerol, with a 0.25% DMSO to achieve sufficient solubility, at different temperatures (Figure 1) because the viscosity of this solvent changed from η = 1412 cP at 20 °C to η = 32 cP at 80 °C [60]. These experiments revealed a fluorescence quantum yield at a higher viscosity, i.e., *Φ*_fl_ = 0.03 at 20 °C, that decreased at a higher temperature (*Φ*_fl_ = 0.01 at 80 °C). This observation indicated additional pathways for a nonradiative deactivation of the excited state by conformational changes, for example, by torsional motions of the substituents or the more flexible C5-C6 ethano unit. However, since the fluorescence in solvents with low viscosity, such as 1-PrOH (*Φ*_fl_ = 0.04, *η* = 2.18 cP) [61] or MeCN (*Φ*_fl_ = 0.03, *η* = 0.36 cP) [61], is comparable to the one in glycerol at 20 °C, the fluorescence obviously depends not only on the viscosity.

### 2.3. Photometric DNA Titrations

**Figure 2:**
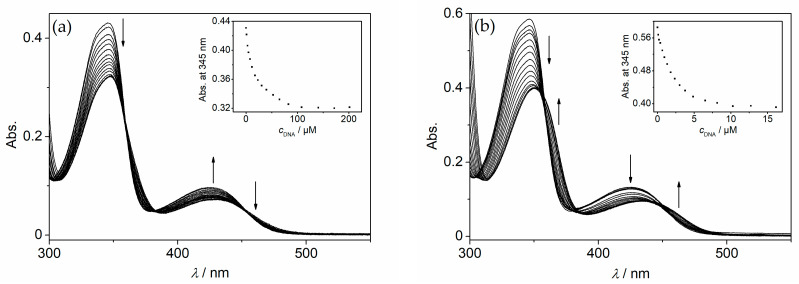
Photometric titration of **2** (*c* = 20 μM) with ct DNA (**a**) in a BPE buffer (*c*_Na+_ = 16 mM, pH 7.0, with 5% *v*/*v* DMSO) and with **a2** (**b**) in a K-phosphate buffer (*c*_K+_ = 110 mM, pH 7.0, with 5% *v*/*v* DMSO). The arrows indicate the changes in absorption upon the addition of DNA. Inset: Plot of the absorption at 345 nm versus DNA concentration.

The binding properties of derivative **2** with double-stranded calf thymus (ct) DNA and G4-DNA were investigated by photometric titrations. For this purpose, a small series of biologically relevant quadruplex-forming oligonucleotides was selected, namely the parallel **c-myc** (d[AG_3_AG_3_CGCTG_3_AG_2_AG_3_]) and **c-kit** (d[TGA(G_3_TG_3_TA)_2_]), and the mixed parallel/antiparallel **a2** (d[(ACAG_3_TGT)_2_]), which are found in the promoter regions of myc, kit and insulin [11,12,13,14], as well as **22AG** (d[A(T_2_AG_3_)_3_G_3_]), which is located in the single-stranded DNA overhang of human telomers and forms a hybrid antiparallel–parallel conformation [15]. As a general trend, a continuous decrease and a red shift of the absorption bands at 346 nm and 424 nm were observed upon addition of DNA (Figure 2, Figure S3). Interestingly, the red shift was slightly more pronounced in the presence of G4-DNA (∆*λ* = 10–12 nm; ∆*ν* = 543–649 cm^−1^) as compared to ct DNA (∆*λ* = 6 nm; ∆*ν* = 329 cm^−1^). Moreover, no isosbestic points were observed during the titrations, which usually indicates different binding modes at varying ligand–DNA ratio (*LDR*). The resulting binding isotherms from the photometric titrations were used to determine the corresponding binding constants *K*_b_ (Table 2, Figure S5) [62]. Accordingly, the binding constant of the ligand **2** with ct DNA was slightly lower (*K*_b_ = 2.2 × 10^5^ M^−1^) than the ones with G4-DNA (*K*_b_ = 5.0–6.9 × 10^5^ M^−1^), and these values are in the same range as the binding parameters of berberine (**1a**) with ct DNA (*K*_b_ = 9.7 × 10^4^ M^−1^) [38] and **22AG** (*K*_b_ = 4.5 × 10^5^ M^−1^) [28]. The relatively high affinity of derivative **2** to DNA is somewhat surprising because the introduction of negatively charged substituents is commonly believed to decrease the affinity of a DNA ligand. For example, the introduction of phosphate groups to known DNA binders, such as naphthalimide, napthaldiimide, or diaminomitosene [47,48,49], leads to a significantly decreased affinity to DNA as compared to the parent compounds, presumably due to the electrostatic repulsion between negatively charged phosphate substituent and the negatively charged DNA backbone. At the same time, anions have been shown to bind to DNA via specific associations with the DNA bases, and a zwitterionic anthraquinone has been reported to bind to DNA with the same affinity as the corresponding cationic system [50]. Thus, the berberine **2** represented one of the rare examples of ligands with anionic substituents that bind with sufficient affinity to duplex DNA. At the same time, the affinity toward G4-DNA was in good agreement with the one of the few already known anionic G4-DNA ligands [51,52,53,54].

### 2.4. Thermal DNA Denaturation Experiments

The stabilization of different G4-DNA forms toward thermally induced unfolding via association with ligand **2** was examined by thermal DNA-denaturation experiments. For that purpose, the melting temperature, *T*_m_, of the dye-labeled quadruplex-forming oligonucleotides **F21T**, **Fa2T**, **FmycT**, and **FkitT** was determined using fluorescence spectroscopy (Table 2, Figures S6 and S7) [63]. The induced shifts of the melting temperature, ∆*T*_m,_ indicated that ligand **2** slightly stabilized the quadruplex forms **F21T** (∆*T*_m_ = 3.6 °C, at *LDR* = 5) and **FmycT** (∆*T*_m_ = 2.2 °C), and has essentially no effect on the oligonucleotides **FkitT** (∆*T*_m_ = 1.0 °C) and **Fa2T** (∆*T*_m_ = 0.6 °C). Notably, the ∆*T*_m_ values were, in most cases, not affected by the presence of a potentially competitive double-stranded DNA as formed by the self-complementary oligonucleotide **ds26** (**F21T**/**ds26**: ∆∆*T*_m_ = –0.4 °C; ^FRET^S = 0.89, **FkitT**/**ds26**: ∆∆*T*_m_ = 0.0 °C; ^FRET^S = 1.00, **Fa2T**/**ds26**: ∆Δ*T*_m_ = −0.2 °C; ^FRET^S = 0.67) (Figures S6 and S7), which revealed a selective association of the ligand with the G4-DNA structures in the mixtures of both oligonucleotides. Only the stabilization of oligonucleotide **FmycT** was affected marginally by the presence of **ds26** (**FmycT**/**ds26**: ∆∆*T*_m_ = −1.3 °C; ^FRET^S = 0.41).

### 2.5. CD and LD Spectroscopy

**Figure 3:**
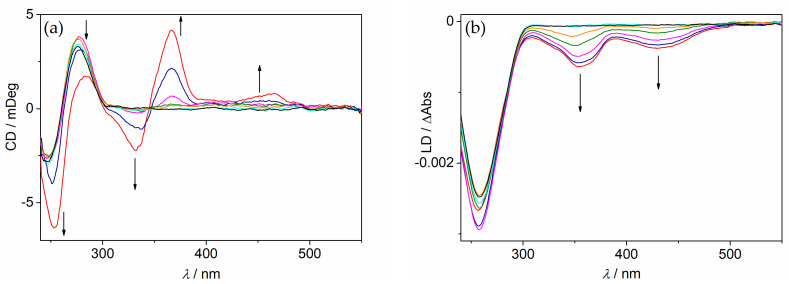
CD spectra (**a**) and LD spectra (**b**) of ct DNA (c_DNA_ = 20 μM) in the absence and presence of **2** at *LDR* = 0 (black), 0.05 (cyan), 0.2 (orange), 0.5 (green), 1.0 (magenta), 1.5 (blue), and 2.0 (red) in a BPE buffer solution (10 mM, pH 7.0; with 5% *v*/*v* DMSO). The arrows indicate the changes in absorption with increasing *LDR*.

Circular dichroism (CD) spectroscopy and flow linear dichroism (LD) spectroscopy were used to examine the interactions of ligand **2** with double-stranded ct DNA in more detail (Figure 3, Figure S8). Upon the addition of ligand **2**, the CD signal of ct DNA at 278 nm continuously decreased and the intensity of the negative signal at 258 nm strongly increased at *LDR* = 2.0, which may be caused by a strong overlapping induced CD (ICD) signal of the absorption band of **2** at 290 nm or by changes of the secondary structure of the DNA during complex formation [64,65]. At the same time, a positive ICD signal developed with a maximum of 430 nm together with a strong bisignate ICD band with zero transition at the absorption maximum of **2** at 345 nm at *LDR* = 1.0–2.0, which indicates the aggregation of the ligand along the DNA backbone at high *LDR* [64,65]. To assess whether aggregation occurs exclusively in the presence of ct DNA, the absorption of a more concentrated solution of **2** (*c* = 500 µM) in a buffer solution was recorded at different temperatures (Figure S2). The absorption of the ligand did not change at different temperatures, which indicated that derivative **2** does not aggregate in the absence of DNA even at these relatively high concentrations.

The flow LD spectra of ct DNA-ligand mixtures revealed negative LD bands with peaks at 354 nm and 430 nm upon addition of the ligand at *LDR* = 0–0.5 (Figure 3B). Such negative LD bands originate from a coplanar alignment of the ligand relative to the DNA bases and prove the intercalation of the ligand into ct DNA as the dominant binding mode at low *LDR* [66] as it has already been found for the parent ligand **1a** [67]. Other than derivate **2**, however, **1a** does not exhibit ICD signals under identical conditions [68], pointing to a different binding mode, specifically a different orientation within the intercalation pocket as compared to ligand **2**. In the case of the intercalated berberine (**1a**), the π stacking of the isoquinolinium part with the DNA bases has been proposed, whereas the benzodioxole unit is accommodated in the groove [67]. Due to the steric hinderance and the negative charge of the phosphate substituent, this binding mode is rather improbable for ligand **2**. Instead, in this case the benzodioxole unit more likely intercalates into the binding pocket and the bulky isoquinolinium-phosphate part points into the groove. In this particular binding mode, the negatively charged phosphate group has the ability to interact with amino groups of adenine, guanine, or cytosine via hydrogen bridging as already reported for sulfate or phosphate ions [43,44,45,46]. However, due to the strong electrostatic repulsion between neighboring ligands, the number of available intercalation sites is limited, leading to aggregation along the DNA backbone as additional binding mode at high *LDR.* Likewise, a known zwitterionic anthraquinone has been assumed to bind by outside-edge binding to DNA [50], which is in agreement with the aggregation of **2** at high *LDR*. Nevertheless, as a notable difference the ligand **2** also intercalates into DNA as confirmed by the LD spectroscopic investigations.

**Figure 4:**
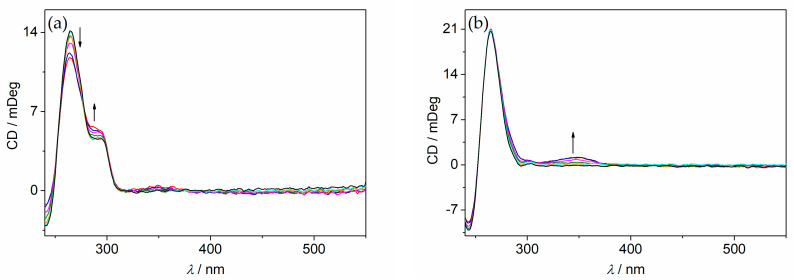
CD spectra of **a2** (**a**, c_DNA_ = 20 μM) and **c-myc** (**b**, c_DNA_ = 20 μM) in the absence and presence of **2** at *LDR* = 0 (black), 0.05 (cyan), 0.2 (orange), 0.5 (green), 1.0 (magenta), 1.5 (blue), and 2.0 (red) in a K-phosphate buffer (*c*_K+_ = 110 mM, pH 7.0, with 5% *v*/*v* DMSO). The arrows indicate the changes in absorption with increasing *LDR*.

**Figure 5:**
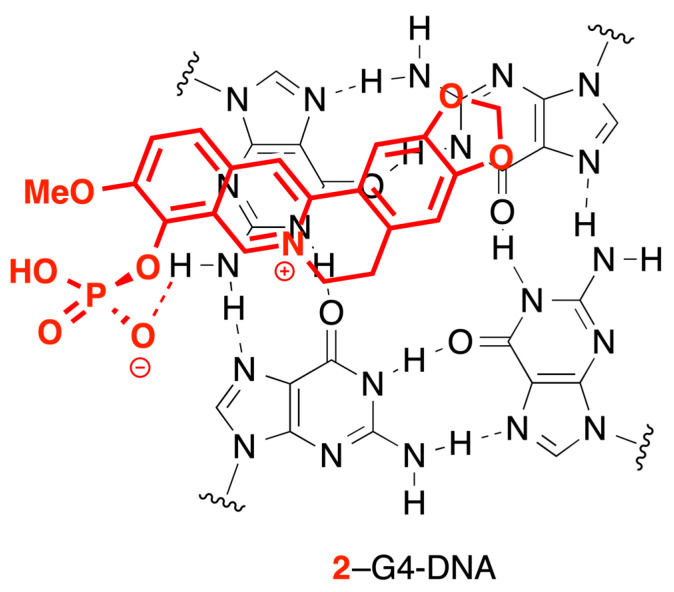
The proposed association of ligand **2** at the terminal quartet of G4-DNA **c-myc** and **c-kit**.

The binding interactions of ligand **2** with G4-DNA were also examined with CD spectroscopy (Figure 4). Upon the addition of **2** to oligonucleotide **a2**, a decrease of the CD signal at 265 nm and an increase of the shoulder at 290 nm were observed. Although these changes were only marginal, they may originate from a shift of the equilibrium from the parallel (260 nm) to the antiparallel (295 nm) conformation of the quadruplex [69,70]. In contrast, the characteristic DNA signals of **22AG**, **c-kit** and **c-myc** only fluctuated a bit or did not even change at all, which indicated that the original conformation of these oligonucleotides remained intact upon association with ligand **2** [71]. Interestingly, only solutions of ligand **2** with antiparallel G4-DNA structures **c-myc** and **c-kit** exhibited ICD bands, whereas the hybrid-type **22AG** and the mixed parallel/antiparallel **a2**, both of which contained loops above the terminal quartet, did not exhibit a significant ICD signal. Thus, weak positive ICD signals were detected at 354 nm after the addition of **2** to oligonucleotides **c-myc** and **c-kit**, which corresponded to the short-wavelength absorption band of the ligand. Since positive ICD signals are caused by a nondegenerative coupling of orthogonal transition dipole moments of the ligand and the DNA bases [70,72], the ICD can be used to deduce the relative orientation of the ligand within the binding site. In fact, it had been proposed recently that such a positive ICD band of berberine–adenine conjugates originates from terminal stacking with the ethano unit of the berberine pointing inside the quadruplex and the bulky substituents at the 9 position extending into the groove [29]. Since the latter 9-*O*-substituted berberine derivatives and **2** are assumed to have similar transition dipole moments, it is concluded that the positive ICD signal of **2** also indicates a resembling binding mode with **c-myc** and **c-kit** (Figure 5). In this binding mode, the phosphate substituent protrudes into the groove where it can coordinate by hydrogen bonding to an amino group of the quartet [44]. The intercalation of the ligand is excluded because this binding mode would require a thermodynamically unfavorable unwinding of the quadruplex structure, and it should lead to a significant change of the CD sinature of the G4-DNA, which we did not observe.

In contrast, no ICD signals of ligand **2** were observed in the presence of **22AG** and **a2**, which is often proposed to indicate terminal π stacking [73,74], also in the case of the parent berberine **1a** [28,75]. Without a clear positive or negative ICD signal, however, the orientation of the ligand in the binding site cannot be determined from this result, but the lack of a positive ICD at least shows that the ligand is positioned differently in the binding site of **22AG** and **a2** than with **c-myc** and **c-kit**. This discrepancy may be the result of the different structures of the G4-DNA forms. Specifically, **22AG** and **a2** contain adenine, guanine, and thymine residues close to the terminal quartets [76,77], whereas, for **c-myc** and **c-kit**, fewer binding sites are available at these particular positions. In the case of **c-kit**, only one amino group of an adenine base is accessible, and **c-myc** contains only one free thymine base at one of the terminal quartets [78,79]. Therefore, the π-stacked ligand has further opportunities to bind with the phosphate to the amino substituents in the loops of **22AG** and **a2**. Furthermore, **22AG** contains an adenine triplet above the terminal G-quartet, which offers an additional binding site for the phosphate group because it has been shown that anions bind well to the adenine quartet [45,46].

### 2.6. Fluorimetric Titrations

**Figure 6:**
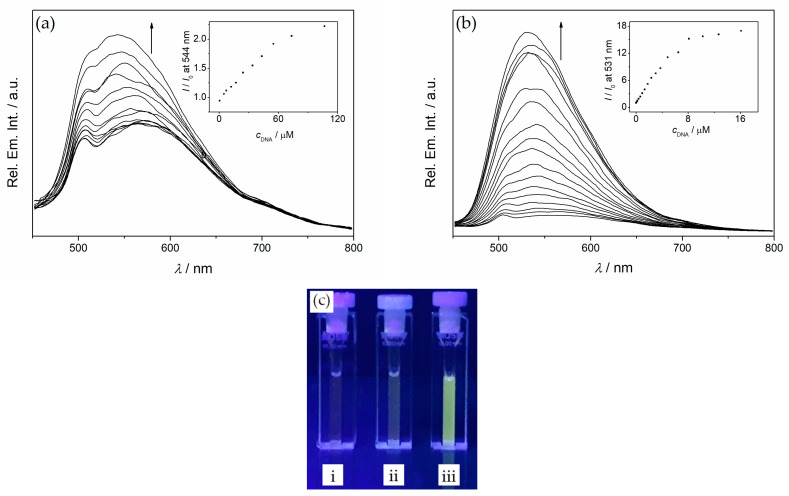
Fluorimetric titration of **2** (*c* = 20 μM, *λ*_ex_ = 430 nm) with ct DNA (**a**) in a BPE buffer (*c*_Na+_ = 16 mM, pH 7.0, with 5% *v*/*v* DMSO) and with **a2** (**b**) in a K-phosphate buffer (*c*_K+_ = 110 mM, pH 7.0, with 5% *v*/*v* DMSO); *λ*_ex_ = 430 nm. The arrows indicate the changes in absorption upon addition of DNA. Inset: Plot of the relative fluorescence intensity versus *c*_DNA_; (**c**) pictures of the emission color of **2** in a K-phosphate buffer (i) and in the presence of ct DNA (ii) and **22AG** (iii) (*λ*_ex_ = 360 nm).

Upon the addition of DNA, the emission of ligand **2**, which is hardly detectable in aqueous buffer solution, continuously increased and emission bands between 530 nm (**22AG**) and 544 nm (ct DNA) developed (Figure 6, Figure S4). Most notably, upon the addition of G4-DNA, a clear light-up effect was observed (*Φ*_fl_ = 0.01), whereas the emission after the addition of ct DNA still remained very low (*Φ*_fl_ < 0.01). Since the quenching of the fluorescence of **2** in an aqueous solution is mainly caused by a CT and conformational relaxation (see above), the partial suppression of both deactivation channels is proposed to cause the increased emission intensity of the DNA-bound ligand. Firstly, emission quenching by conformational motions is likely suppressed or at least hindered in the sterically constrained binding sites. Secondly, the deactivation of the excited state by a CT is suppressed in the less polar environment within the DNA binding site [55,56,80,81]. In the case of the complexation of ligand **2** and ct DNA, however, two different binding modes need to be considered. Upon aggregation along the DNA backbone at high *LDR*, the ligand is not completely shielded from the polar aqueous environment, so the excited state is still deactivated by CT. Moreover, the aggregation enables additional deactivation pathways by excitonic coupling or charge transfer between the aggregated molecules [82,83], leading, in combination with the CT, to a very low fluorescence of the DNA-bound aggregate. Moreover, upon intercalation into the duplex DNA, the conformational freedom is hindered and the ligand is somewhat shielded from the buffer solution. Overall, the combination of these effects upon binding of **2** to ct DNA results in a small but still significant increase in the emission intensity.

The more pronounced increase in the emission intensity of the G4-DNA-bound ligand **2** may indicate that the conformational flexibility of the ligand was suppressed more efficiently upon complexation to this DNA form, e.g., by a tight accommodation of the ligand in the binding site. Furthermore, the ligand was shielded from the aqueous environment, thus leading, in combination with the restriction of conformational flexibility, to a more pronounced light-up effect. Interestingly, the complexes of **2** with **c-myc** and **c-kit**, which showed a positive ICD, also exhibited slightly lower fluorescence quantum yields (**c-myc**: *Φ*_fl_ = 0. 008; **c-kit**: *Φ*_fl_ = 0.008) compared to the complexes with **22AG** (*Φ*_fl_ = 0.011) and **a2** (*Φ*_fl_ = 0.010). Although these differences were very small, especially considering the error limit, they may still indicate the different binding modes of **2** to the particular G4-DNA forms (see above), which in turn caused a slightly different extent of fluorescence quenching. 

## 3. Materials and Methods

### 3.1. Equipment

Absorption: Varian Cary 100 Bio with baseline correction. Emission: Varian Cary Eclipse at 20 °C. Cuvettes: Quartz cells (10 mm × 4 mm). NMR spectra: Jeol ECZ 500 (^1^H: 500 MHz, ^13^C: 125 MHz) at 25 °C at 25 °C (DMSO-*d*_6_). NMR spectra were processed with the software MestReNova and referenced to the solvent DMSO-*d*_6_ (δ(DMSO-*d*_5_): ^1^H = 2.50 ppm, ^13^C: δ = 39.5 ppm). Elemental analyses data: HEKAtech EUROEA combustion analyzer, by Rochus Breuer, Organische Chemie I, Universität Siegen. Mass spectrometry (ESI): Finnigan LCQ Deca (U = 6 kV; working gas: Ar; auxiliary gas: N_2_; temperature of the capillary: 200 °C). Circular dichroism (CD) and flow-linear dichroism (LD): Chirascan CD spectrometer, Applied Photophysics. For LD spectra: High Shear Cuvette Cell Accessory (Applied Photophysics). The LD samples were recorded in a rotating cuvette with a shear gradient of 1200 s^−1^. Melting points (uncorrected): BÜCHI 545 (BÜCHI, Flawil, CH). 

### 3.2. Materials

Berberrubine (**1b**) was synthesized according to published procedures [84]. Calf thymus DNA (ct DNA, type I; highly polymerized sodium salt; *ε* = 12824 cm^−1^M^−1^) [85] was purchased from SigmaAldrich (St. Louis, MO, USA) and used without further purification. Oligodeoxyribonucleotides (HPLC purified) d[A(GGGTTA)_3_GGG] (**22AG**), d[(ACAGGGGTGTGGGG)_2_] (**a2**), d[TGAG_3_TG_3_TAG_3_TG_3_TA] (**c-myc**), d[(AG_3_AG_3_CGCTG_3_AG_2_AG_3_)] (**c-kit**), d[CA_2_TCG_2_ATCGA_2_T_2_CGATC2GAT_2_G] (**ds26**), d[fluo-(GGGTAA)_3_GGG-tamra] (**F21T**), d[fluo-(ACAGGGGTGTGGGG)_2_-tamra] (**Fa2T**), d[fluo-TGAG_3_TG_3_TAG_3_TG_3_TA-tamra] (**FmycT**), and d[fluo-(AG_3_AG_3_CGCTG_3_AG_2_AG_3_)-tamra] (**FkitT**) (fluo = fluorescein, tamra = tetramethylrhodamine) were purchased from Metabion Int. AG (Planegg/Martinsried). The concentration of ct DNA is given in base pairs (bp). 

The ct DNA was dissolved in a BPE buffer solution. Solutions of oligonucleotides were prepared in a K-phosphate buffer, heated to 95 °C for 5 min, and cooled slowly to room temperature within 4 h. K-phosphate buffer: 25 mM K_2_HPO_4_, 60 mM KCl; adjusted with 50 mM KH_2_PO_4_, 60 mM KCl to pH 7.0; BPE (biphosphate EDTA) buffer: 6.0 mM Na_2_HPO_4_, 2.0 mM NaH_2_PO_4_, 1.0 mM Na_2_EDTA; pH 7.0. All buffer solutions were prepared from purified water (resistivity 18 MΩ cm) and biochemistry-grade chemicals. The buffer solutions were filtered through a PVDF membrane filter (pore size 0.45 μm) prior to use.

### 3.3. Synthesis

10-*Methoxy*-5,6-*dihydro*-[1,3]*dioxolo*[4,5-*g*]*isoquinolino*[3,2-*a*]*isoquinolin*-7-*ium*-9-*yl hydrogen phosphate*. A mixture of **1b** (321 mg, 1.00 mmol) and Et_3_N (153 µl) in CH_2_Cl_2_ (40 mL) was cooled to 0 °C and POCl_3_ (109 µl, 183 mg, 120 µmol) was added. The reaction mixture was stirred for 30 min at r.t. and the formed precipitate was filtered. The yellow crude was dissolved in an aqueous HCl solution (1 M, 20 mL) and the aqueous solution was extracted with CH_2_Cl_2_ (3 × 30 mL). The organic layer was washed with water (2 × 20 mL) and brine (20 mL) and dried with Na_2_SO_4_. The drying agent was filtered off and the solvent was removed by distillation to leave the product **2** as a yellow amorphous solid (177 mg, 441 µmol, 44%). To obtain an analytically pure sample, **2** was recrystallized from MeOH/EtOAc into product **2** as yellow needles; m.p. > 246 °C (dec.), ^1^H-NMR (500 MHz, DMSO-*d*_6_, Figure S9): δ = 3.21 (t, ^3^*J* = 6 Hz, 2H, 5-H), 3.87 (s, 3H, OCH_3_), 4.86 (t, ^3^*J* = 6 Hz, 2H, 6-H), 6.17 (s, 2H, OCH_2_O), 7.10 (s, 1H, 4-H), 7.81 (s, 1H, 1-H), 8.02 (d, ^3^*J* = 9 Hz, 1H, 12-H), 8.18 (d, ^3^*J* = 9 Hz, 1H, 11-H), 8.96 (s, 1 H, 13-H), 10.12 (s, 1H, 8-H). ^13^C NMR (125 MHz, DMSO-*d*_6_, Figure S10): δ = 26.5 (C5), 55.9 (C6), 57.1 (OCH_3_), 102.1 (OCH_2_O), 105.5 (C1), 108.5 (C4), 120.3 (C13), 120.5 (C13b), 122.1 (C8a), 123.5 (C12), 127.1 (C11), 130.5 (C4a), 133.0 (C12a), 137.0 (C13a), 138.6 (C9), 146.6 (C8), 147.7 (C2), 149.8 (C3), 150.9 (C10). MS (ESI^+^): *m*/*z* (%) = 302 (100) [M + H]^+^. El anal for C_19_H_16_NO_7_P × 1.5 H_2_O: calcd. (%) C 53.28, H 4.47, N 3.27 found (%) C 53.67, H 4.21, N 3.04.

## 4. Conclusions

In summary, a novel berberine-based DNA ligand with a phosphate substituent was synthesized that binds with high affinity to ct DNA and G4-DNA. With these particular properties, this derivative represents one of the few reported phosphate-substituted DNA ligands. Most notably, ligand **2** binds preferentially to G4-DNA relative to duplex DNA and exhibits a selective light-up effect upon binding to quadruplex-forming oligonucleotides, whereas the emission intensity increases to a much lesser extent upon binding to the double-stranded ct DNA. The significantly increased emission intensity in **2** upon association with G4-DNA is mainly caused by the binding mode, namely terminal π stacking, which shields the ligand from the polar aqueous environment and suppresses fluorescence quenching by a CT. Considering the selectivity of the light-up effect, this ligand has the potential to be employed for the fluorimetric detection of G4-DNA structures in biological samples.

Overall, it was demonstrated that a phosphate unit does not necessarily decrease the affinity of a DNA-binding ligand and that this substituent may even support the selective association with G4-DNA. Therefore, we propose that this observation may be used as starting point for the development of a novel type of DNA-binding ligand by combining other established cationic DNA intercalators, e.g., ethidium, acridinium, or quinolizinium, with a phosphate group.

## Figures and Tables

**Table 1:** Absorption and emission properties of derivatives **1a** and **2** in different solvents.

Solvent	*λ*_abs_^a^/nm		lg *ε* ^b^	*λ*_fl_^c^/nm		*ν*_abs_ − *ν*_fl_^d^/cm^−1^		*Φ*_fl_^e^	
	2	1a ^f^	2	2	1a ^f^	2	1a ^f^	2	1a ^f^
MeOH	431	429	3.98	558	540	5280	4790	0.01	0.01
EtOH	431	430	3.98	537	536	4580	4600	0.02	0.03
1-PrOH	431		3.94	531		4369		0.04	
MeCN	431	430	3.91	537	537	4579	4630	0.03	0.02
DMSO	430	425	3.97	547	553	4974	5500	0.01	<0.01
Buffer	424		3.92	535		4893		<0.01	

^a^ Long-wavelength absorption maximum; *c* = 20 µM. ^b^ *ε* = Molar extinction coefficient in cm^−1^ M^−1^. ^c^ Fluorescence emission maximum; λ_ex_ = 430 nm. ^d^ Stokes shift: Energy difference between absorption and emission maximum. ^e^ Fluorescence quantum yield relative to coumarin 153 in EtOH (*Φ*_fl_ = 0.544). Ref. [59]. ^f^ Ref. [41].

**Table 2:** Binding constants, *K*_b_, and shifts of DNA melting temperature, ∆*T*_m_, of **2** with ct DNA and G4-DNA, and fluorescence quantum yields, *Φ*_fl,_ of the DNA-bound ligands.

DNA	*K*_b_/10^5^ M^−1 b^	*Φ*_fl_ ^c^	∆*T*_m_/°C ^d^
ct DNA	2.2 ± 0.2	<0.01	
**22AG/** **F21T** ^a^	5.0 ± 0.6	0.01	3.6
**c-kit** **/FkitT** ^a^	6.9 ± 0.2	0.01	1.0
**a2**/**Fa2T**^a^	6.7 ± 0.2	0.01	0.6
**c-myc/FmycT** ^a^	5.9 ± 0.4	0.01	2.2

^a^ Dye-labeled oligonucleotides: **F21T** = fluo-G_3_(TTAG_3_)_3_-tamra, **Fa2T** = fluo-(ACAG_3_TGT)_2_-tamra, **FmycT** = fluo-TGAG_3_TG_3_TAG_3_TG_3_TA-tamra, **FkitT** = fluo-AG_3_AG_3_CGCTG_3_AG_2_AG_3_-tamra, fluo = fluorescein, tamra = tetramethylrhodamine ^b^ Determined from photometric titrations (cf. Figure S5). ^c^ Fluorescence quantum yield relative to coumarin 153 in EtOH (*Φ*_fl_ = 0.544, Ref. [59]). ^d^ Determined from fluorimetric analysis of dye-labeled oligonucleotides, *LDR* = 5; *c*_DNA_ = 0.2 mM (in oligonucleotides); KCl–LiCl–cacodylat buffer *c*_K+_ = 10 mM, *c*_Na+_ = 10 mM, *c*_Li+_ = 90 mM, pH 7.0) λ_ex_ = 470 nm; λ_em_ = 515 nm; estimated error: ±0.5 °C.

## Data Availability

Data is available from the authors.

## Supplementary Materials

The following items are available online, Figure S1: Absorption and emission spectra of **2**, Figure S2: Absorption spectra of **2** in a BPE buffer at different temperatures, Figure S3: Photometric titration of **2** with **22AG**, **c-kit** and **c-myc**, Figure S4: Fluorimetric titration of **2** with **22AG**, **c-kit** and **c-myc**, Figure S5: Fitting curves of binding isotherms from photometric titrations of **2** with DNA, Figure S6: Normalized changes in emission intensity of the dye-labeled oligonucleotides upon the addition of **2**, Figure S7: Induced shift in the melting temperature, ∆*T*_m_, of dye-labeled oligonucleotides upon addition of **2**, Figure S8: CD spectra of oligonucleotides **22AG** and **c-kit** in the absence and presence of **2**, Figure S9: ^1^H-NMR spectrum of **2**, Figure S10: ^13^C-NMR spectrum of **2**. References [30,59,62,86,87] are cited in the supplementary materials.

## Sample Availability

Samples of the compound **2** are available from the authors.
