# Supplementary material for: Berberrubine Phosphate: A Selective Fluorescent Probe for Quadruplex DNA
Source: Molecules. 2021 Apr 28;26(9):2566. doi: 10.3390/molecules26092566 (PMC8124163; doi:10.3390/molecules26092566)
Supplement: Supplementary file 1 [file molecules-26-02566-s001.zip › molecules-1177328-supplementary.pdf]

Berberrubine phosphate: A selective Fluorescent Probe for Quadruplex DNA

Peter Jonas Wickhorst and Heiko Ihmels

Department of Chemistry – Biology, University of Siegen, Center of Micro- and Nanochemistry and Engineering (*Cμ*), Adolf-Reichwein-Str. 2, 57068 Siegen, Germany.

Supporting Information

Table of Contents

1. Absorption and emission spectra S2

2. Determination of fluorescence quantum yields S3

4. Photometric and fluorimetric DNA titrations S4

5. Thermal DNA-denaturation experiments S7

6. CD- and LD-spectroscopic analysis S8

7. NMR spectra S9

8. References S10

1. Absorption and emission spectra

Solutions were prepared for each measurement from stock solutions of the derivative **2** in MeOH (*c* = 1.0 mM). Aliquots of the stock solution were thoroughly evaporated under a stream of nitrogen, and the residue was redissolved in the respective solvent or solvent mixture.

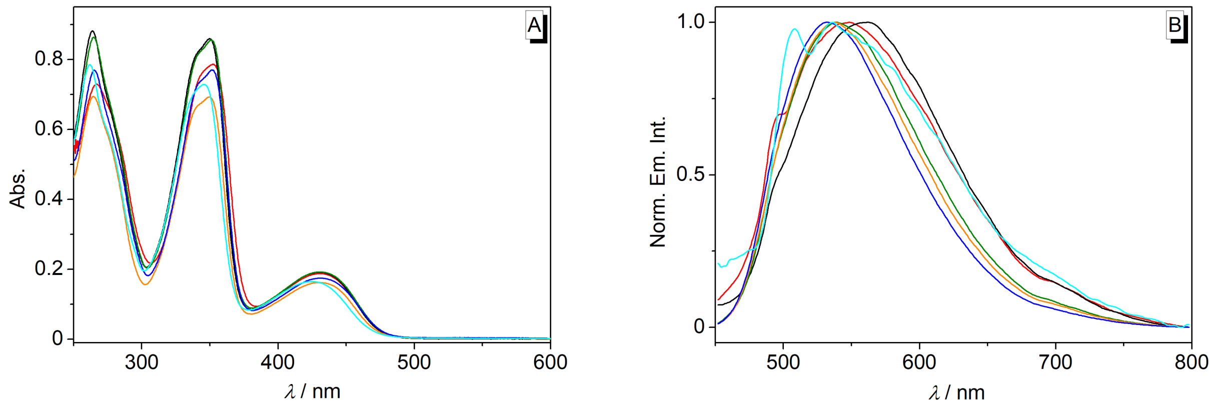

Figure S1. Absorption (A) and emission spectra (B) of **2** (c = 20 μM) in different solvents (red: DMSO, black: MeOH, cyan: aq. buffer, orange: MeCN, green: EtOH, blue: 1-PrOH; buffer: 16 mM BPE buffer solution at pH 7, λ_{ex} = 430 nm).

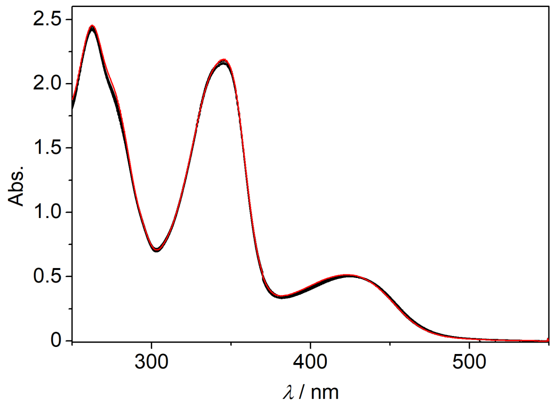

Figure S2. Absorption spectra of **2** (c = 20 μM) in BPE buffer at different temperature (black: 20–70 °C, red: 80°C.

2. Determination of fluorescence quantum yields

Solutions were prepared for each measurement as described above from stock solutions of **2** in MeOH (c = 1.0 mM). For the detection of fluorescence spectra, the excitation and emission slits were adjusted to 5 nm, and the excitation wavelengths were fixed to 340 nm. The relative fluorescence quantum yields, $\Phi_{fl,}$ of **2** were determined under identical conditions (detection wavelength, excitation wavelength, detector voltage, slit bandwidths, collection rate). The quantum yield, Φ_{fl}, was determined according to equation 1.

$$\Phi_{\text{fl, X}} = \frac{F_X A_S}{F_S A_X} \cdot \frac{n_X^2}{n_S^2} \cdot \Phi_{\text{fl, S}} \qquad \text{(eq. 1)}$$

where, the indices X and S indicate the analyte (X) and standard (S) solution.

Φ_{fl} = Fluorescence quantum yield.

F = Integral of the emission band.

A = Absorbance at the excitation wavelength.

n = Refraction index of the solution.

Measurements were performed with coumarin 153 in ethanol as standard [Φ = 0.544] 1.

The estimated error is ca. 10% of the given values.

4. Photometric and fluorimetric DNA titrations

Spectrophotometric and spectrofluorimetric titrations with ct DNA and quadruplex-forming oligonucleotides were performed according to published procedure [2]. All titrations were performed with 5% v/v DMSO to ensure a sufficient solubility of the ligand.

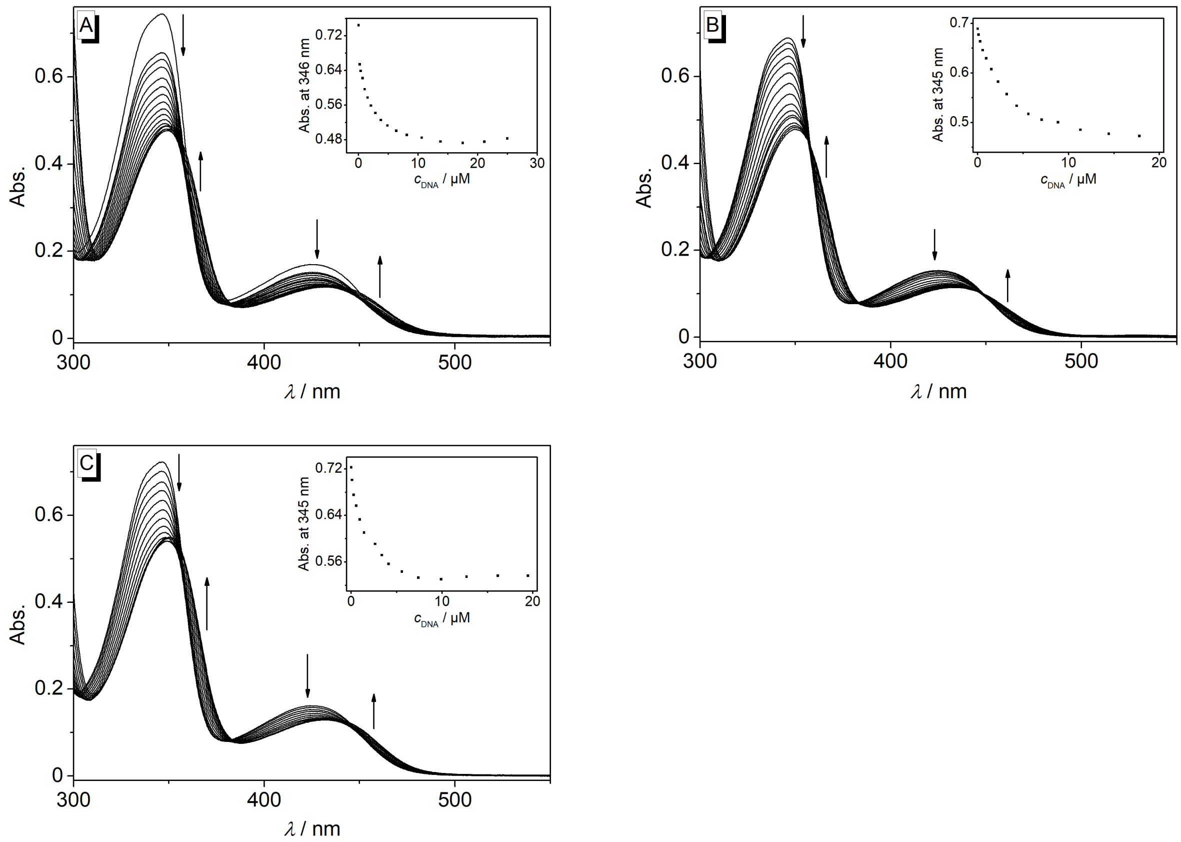

Figure S3. Photometric titration of **2** (c = 20 µM) with **22AG** (A), **c-kit** (B) and **c-myc** (C) in K-phosphate buffer (c_{K+} = 110 mM, pH 7.0, with 5% v/v DMSO). The arrows indicate the changes of the absorption bands upon addition of DNA. Inset: Plot of absorption at 345 nm *versus* DNA concentration.

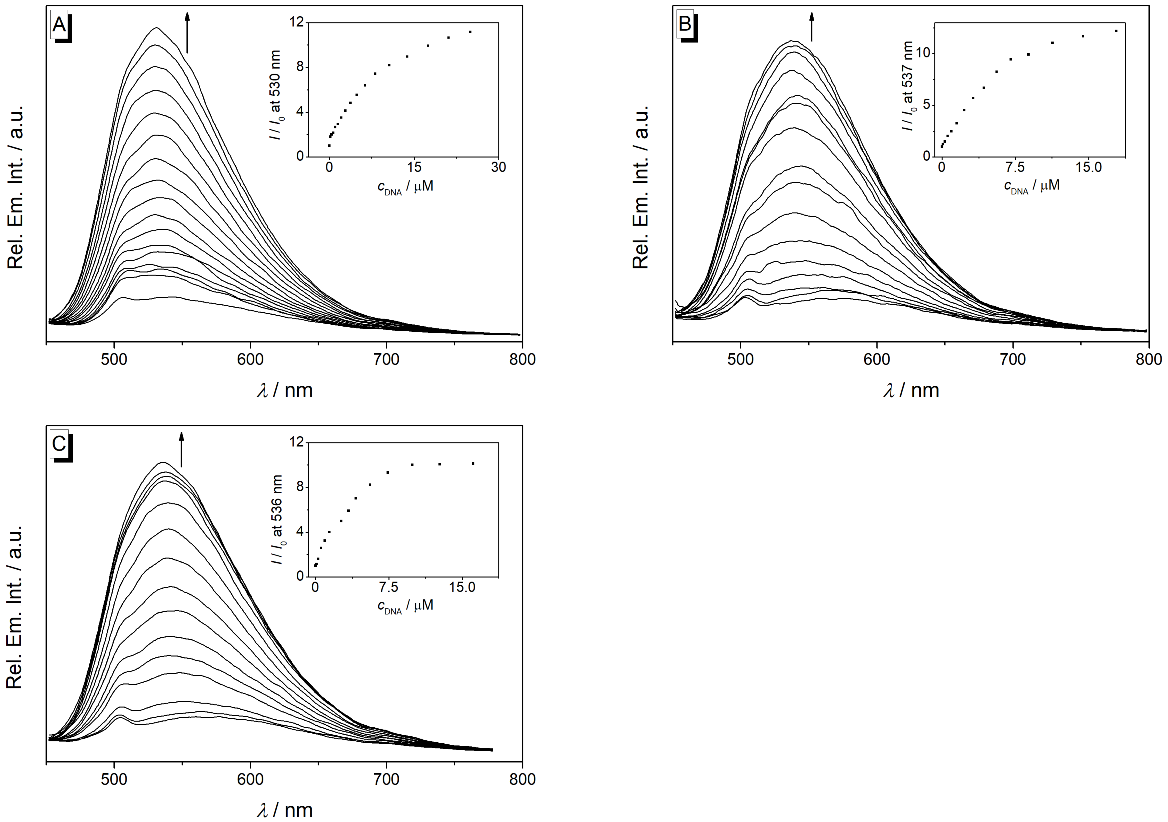

Figure S4. Fluorimetric titration of **2** (c = 20 µM) with **22AG** (A), **c-kit** (B) and **c-myc** (C) in K-phosphate buffer (c_{K+} = 110 mM, pH 7.0, with 5% v/v DMSO); λ_{ex} = 430 nm. The arrows indicate the changes of the emission bands upon addition of DNA. Inset: Plot of the relative fluorescence intensity *versus* c_{DNA}.

The binding constants, K_b, were determined from binding isotherms resulting from the photometric titration (Figure S6) and fitting of the experimental data to the theoretical model according to equation 2 [3].

$$\frac{I}{I_0} = 1 + \frac{Q-1}{2}\left(A + xn + 1 - \sqrt{(Q + xn + 1)^2 - 4xn}\right) \qquad \text{(eq. 2)}$$

$Q = I / I_0$ = Minimal absorbance in the presence of excess ligand

n = Number of independent binding sites per DNA

$A = 1 / (K_b \times c_{Ligand})$

$x = c_{DNA} / c_{Ligand}$

Standard derivations (SD) of K_b values were calculated according to equation 4.

$$\mathrm{SD}\ (K_b) = \{(\mathrm{SD\ of}\ A\ /\ A)\ /\ A\}\ /\ c_{\mathrm{Ligand}} \qquad \text{(eq. 4)}$$

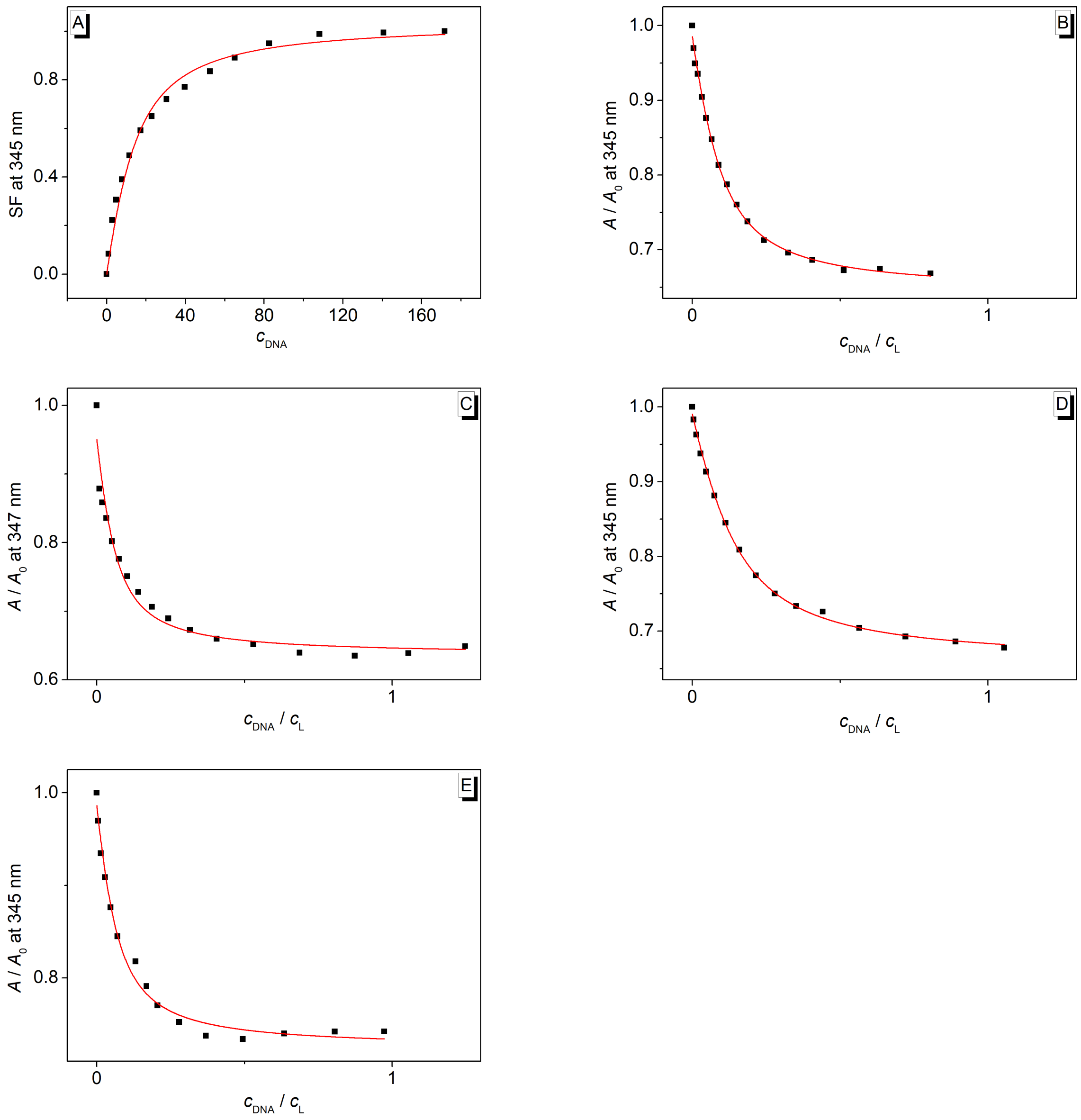

Figure S5. Fitting curves of binding isotherms from photometric titrations of **2** with ct DNA (A), **a2** (B), **22AG** (C), **c-kit** (D) and **c-myc** (E) for the determination of binding constants (K_b). Red lines represent the best fits of the experimental data to the theoretical model.

5. Thermal DNA-denaturation experiments

Thermal DNA-denaturation experiments with the dye-labeled quadruplex-forming oligonucleotides **F21T**, **Fa2T**, **FmycT**, and **FkitT** were performed according to published procedure [4]. ^{FRET}S values were calculated according to equation 3 [5].

$$^{FRET}S = \Delta T_{m,\ G4\text{-}DNA+ds26} / \Delta T_{m,\ G4\text{-}DNA} \qquad \text{(eq. 3)}$$

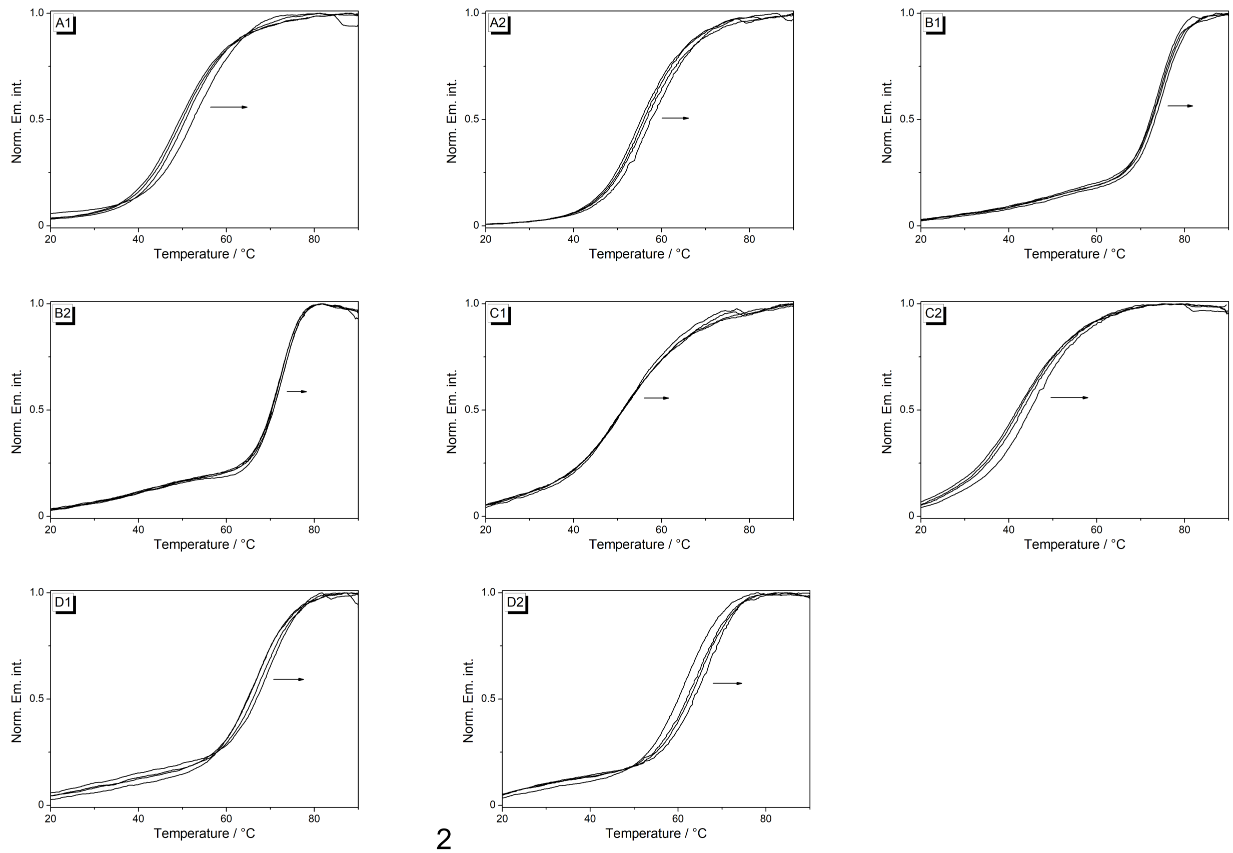

Figure S6. Normalized changes of emission intensity of the dye-labeled oligonucleotides **F21T** (A), **Fa2T** (B), **FkitT** (C), **FmycT** (D) (0.2 µM, λ_{ex} = 470 nm) at 515 nm between 20 °C and 90 °C on addition of ligand **2** in the absence (1) and presence (2) of **ds26**. Thermal denaturation experiments were performed in aqueous KCl-LiCl-Na-cacolydate buffer (c_{K+} = 10 mM, c_{Na+} = 10 mM, c_{K+} = 90 mM, pH 7.2) at different ligand-DNA ratios (0, 1.3, 2.5, 5.0).

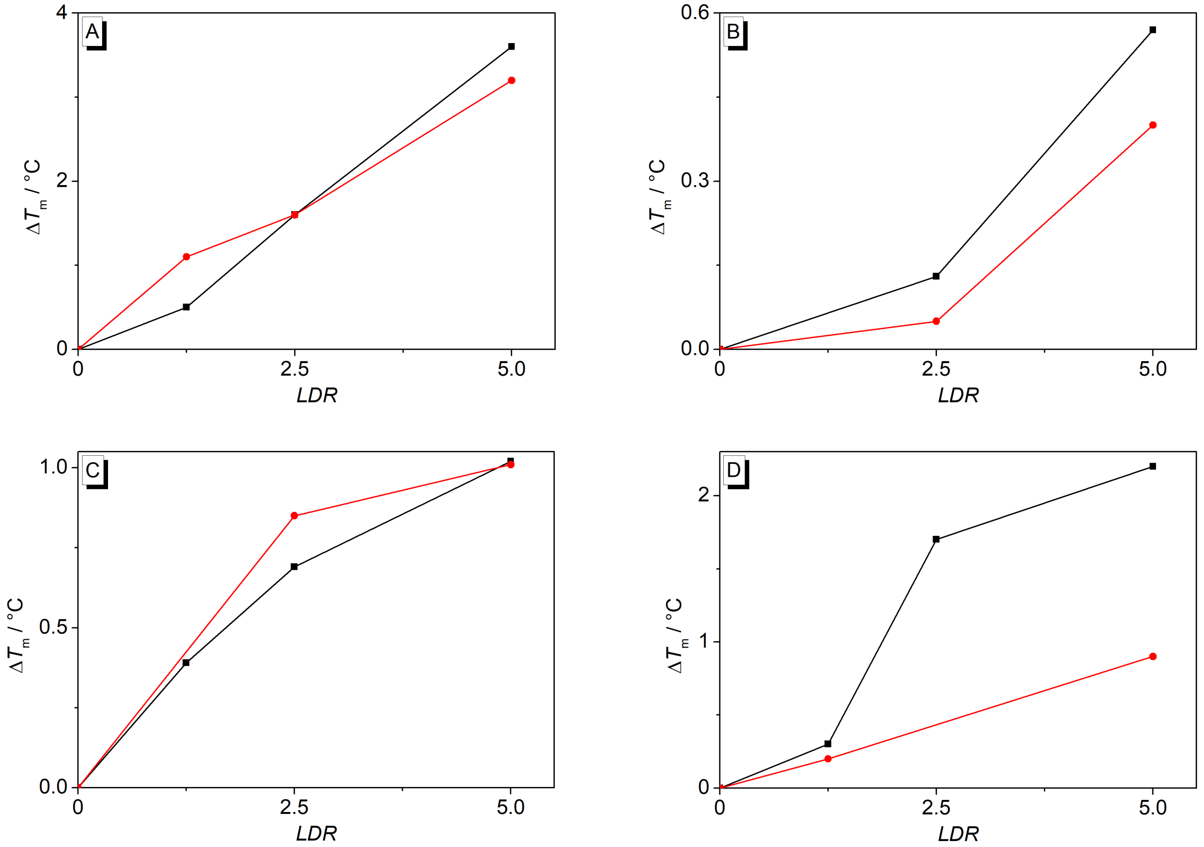

Figure S7. Induced shift of the melting temperature, ΔT_m, of **F21T** (A), **Fa2T** (B), **FkitT** (C) and **FmycT** (D) (c = 50 µM) upon addition of ligand **2** in the absence (black) and presence (red) of **ds26**. Thermal DNA-denaturation experiments were performed in aqueous KCl-LiCl-Na-cacolydate buffer (c_{K+} = 10 mM, c_{Na+} = 10 mM, c_{K+} = 90 mM, pH 7.2) at different ligand-DNA ratios (0, 1.3, 2.5, 5.0).

6. CD- and LD-spectroscopic analysis

CD and LD spectra were recorded in BPE buffer solution with ct DNA (c_{DNA} = 20.0 µM, with 5% v/v DMSO) and in K-phosphate buffer solution with **22AG, a2**, **c-kit**, **c-myc** (c_{DNA} = 20.0 µM, with 5% v/v DMSO) at different ligand-DNA ratios (*LDR*). The CD and LD measurements with ct DNA were performed at *LDR* = 0, 0.1, 0.2, 0.5, 1.0. CD signals were recorded with band width of 1 nm, recording speed of 1 nm/s and time per data point of 0.5 seconds. All samples were measured after an equilibration time of 1 h.

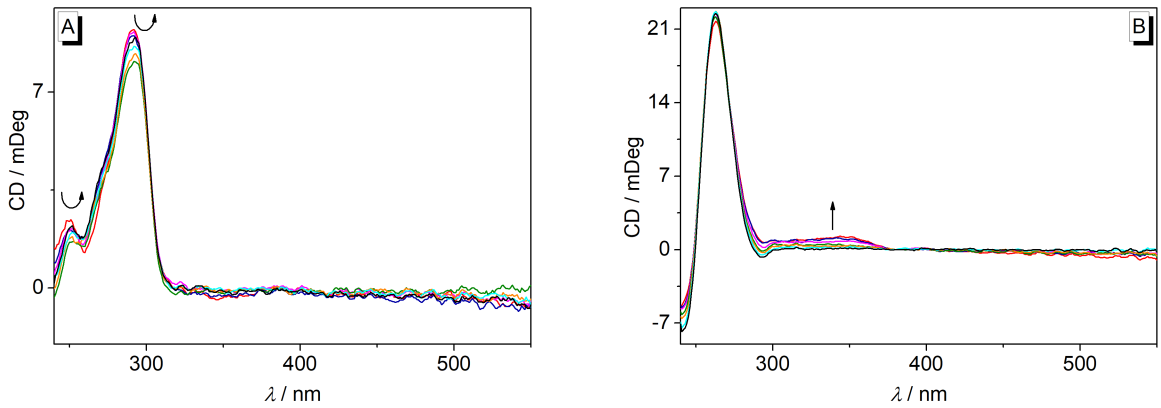

Figure S8. CD spectra of oligonucleotides **22AG** (A) and **c-kit** (B) (c_{DNA} = 20 μM) in the absence and presence of **2** [LDR = 0 (black), 0.05 (cyan), 0.2 (orange), 0.5 (green), 1.0 (magenta), 1.5 (blue), 2.0 (red)] in K-phosphate buffer (c_{K+} = 110 mM, pH 7.0, with 5% v/v DMSO). The arrows indicate the changes of CD bands upon addition of DNA.

7. NMR spectra

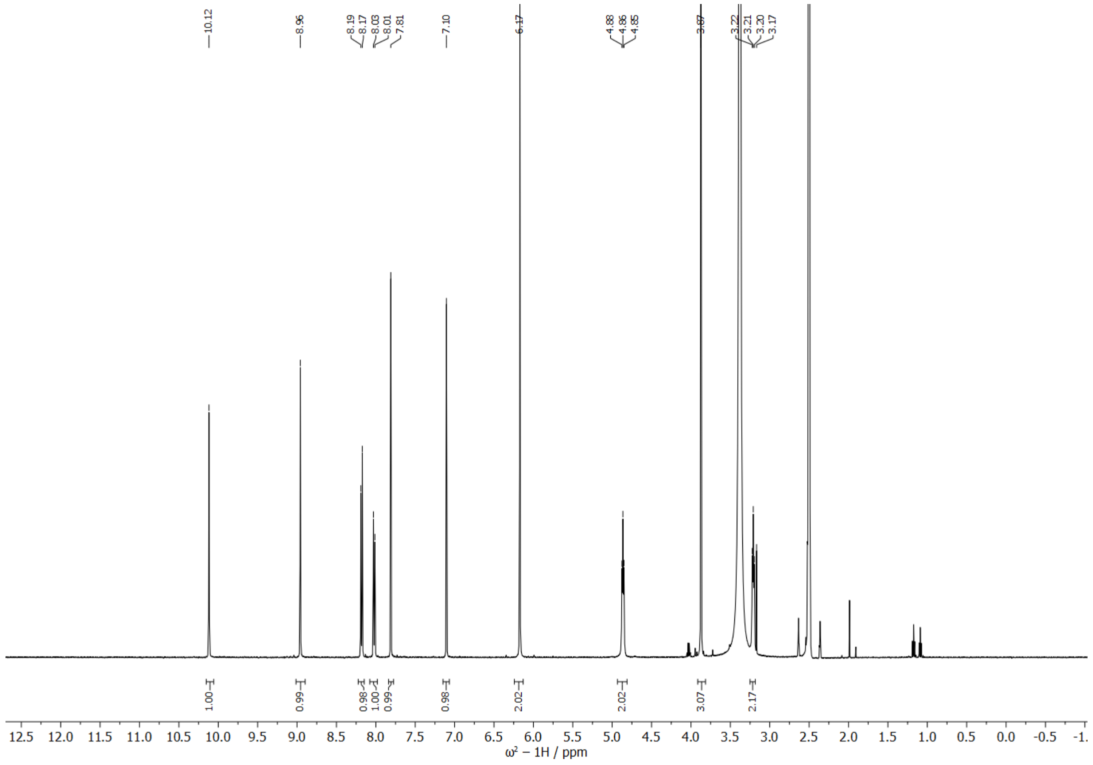

Figure S9. ^{1}H-NMR spectrum (500 MHz) of **2** in DMSO-d_6.

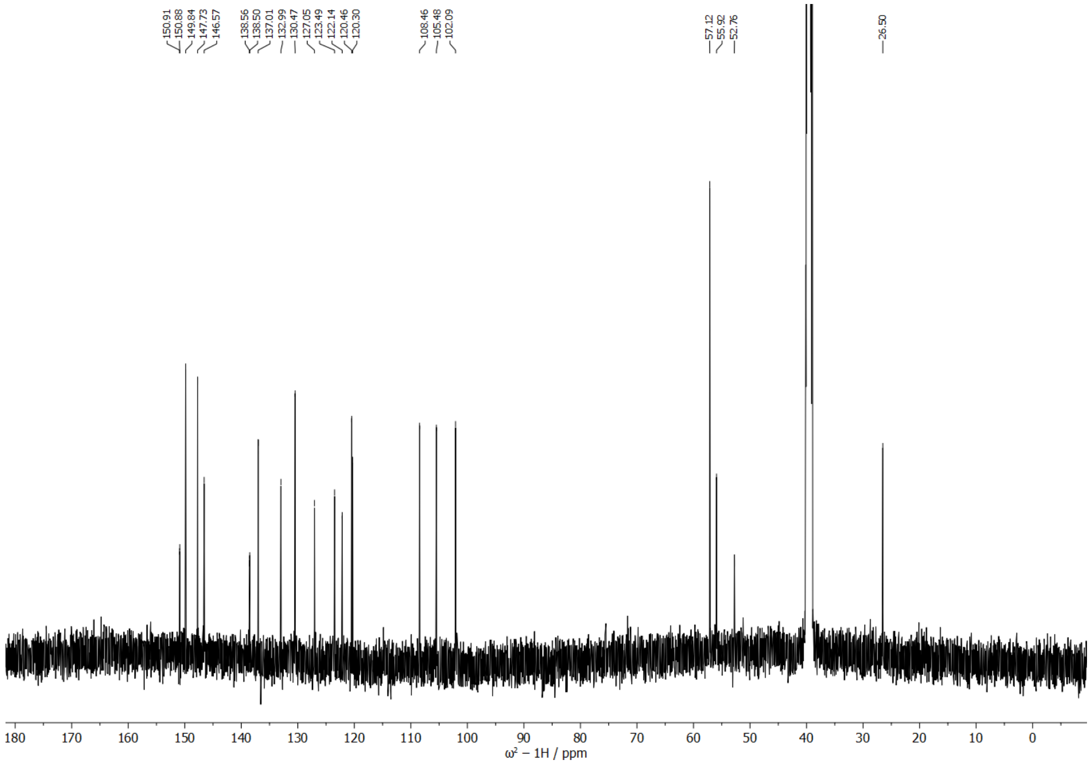

Figure S10. ^{13}C-NMR spectrum (125 MHz) of **2** in DMSO-d_6.

8. References

1. Rurack, K.; Spieles, M. Fluorescence Quantum Yields of a Series of Red and Near-Infrared Dyes Emitting at 600−1000 nm. *Anal. Chem.* **2011**, *83*, pp. 1232–1242.

2. Bortolozzi, R.; Ihmels, H.; Thomas, L.; Tian, M.; Viola, G. 9-(4-Dimethylaminophenyl)-benzo[*b*]quinolizinium: A Near-Infrared Fluorophore for the Multicolor Analysis of Proteins and Nucleic Acids in Living Cells. *Chem. Eur. J.* **2013,** *19,* pp. 8736–8741.

3. Stootman, F. H.; Fisher, D. M.; Rodger, A.; Aldrich-Wright, J. R. Improved curve fitting procedures to determine equilibrium binding constants. *Analyst* **2006**, *131*, pp. 1145–1151.

4. Pithan, P. M.; Decker, D.; Druzhinin, S. I.; Ihmels, H.; Schönherr, H.; Voß, Y. 8-Styryl-substituted coralyne derivatives as DNA binding fluorescent probes. *RSC Adv.* **2017**, *7*, pp. 10660-10667.

5 Duskova, K.; Lejault, P.; Benchimol, E.; Guillot, R.; Britton, S.; Granzhan, A.; Monchaud, D. DNA Junction Ligands Trigger DNA Damage and Are Synthetic Lethal with DNA Repair Inhibitors in Cancer Cells. *J. Am. Chem. Soc.* **2020**, *142*, pp. 424–435.
